# The Dangers of Analyzing Thermographic Radiometric Data as Images

**DOI:** 10.3390/jimaging9070143

**Published:** 2023-07-12

**Authors:** Časlav Livada, Hrvoje Glavaš, Alfonzo Baumgartner, Dina Jukić

**Affiliations:** Faculty of Electrical Engineering, Computer Science and Information Technology Osijek, Josip Juraj Strossmayer University in Osijek, Kneza Trpimira 2B, 31000 Osijek, Croatia

**Keywords:** infrared thermography, radiometric analysis, temperature measurement, image processing, JPEG compression

## Abstract

Thermography is probably the most used method of measuring surface temperature by analyzing radiation in the infrared part of the spectrum which accuracy depends on factors such as emissivity and reflected radiation. Contrary to popular belief that thermographic images represent temperature maps, they are actually thermal radiation converted into an image, and if not properly calibrated, they show incorrect temperatures. The objective of this study is to analyze commonly used image processing techniques and their impact on radiometric data in thermography. In particular, the extent to which a thermograph can be considered as an image and how image processing affects radiometric data. Three analyzes are presented in the paper. The first one examines how image processing techniques, such as contrast and brightness, affect physical reality and its representation in thermographic imaging. The second analysis examines the effects of JPEG compression on radiometric data and how degradation of the data varies with the compression parameters. The third analysis aims to determine the optimal resolution increase required to minimize the effects of compression on the radiometric data. The output from an IR camera in CSV format was used for these analyses, and compared to images from the manufacturer’s software. The IR camera providing data in JPEG format was used, and the data included thermographic images, visible images, and a matrix of thermal radiation data. The study was verified with a reference blackbody radiation set at 60 ${}^{{}^{\circ}}$C. The results highlight the dangers of interpreting thermographic images as temperature maps without considering the underlying radiometric data which can be affected by image processing and compression. The paper concludes with the importance of accurate and precise thermographic analysis for reliable temperature measurement.

## 1. Introduction

All bodies and objects with temperature above 0 K radiate energy in the form of electromagnetic waves. The quantity of energy radiating from a surface as heat can be roughly estimated as proportional to the fourth power of the body or object’s absolute temperature and its emissivity. A naked human eye detects visible radiation of the electromagnetic spectrum that is the result of reflected solar radiation or radiation provided by artificial lights. However, all bodies and most objects in thermal equilibrium radiate in the infrared (IR) part of the spectrum which falls between the visible and microwave regions. Infrared thermography (IRT) is a non-destructive method to bodies/objects that can be used for the detection of such radiation, oftentimes referred to as thermal radiation. A thermographic camera (IR camera) registers radiated heat in the IR spectrum and displays it as a heat pattern in a narrower, visible part of the spectrum. A lens in the camera focuses the emitted IR radiation onto a detector and further the electrical response signal is converted into a digital picture. The digital picture, called a thermograph, is a visual representation of the temperature differences across the surface of the target. Various color patterns or gray shades correspond to different temperature levels of the surface on which the camera was focused on. To analyze the thermograph and quantify temperature differences within, appropriate software is used.

The use of thermography, since the discovery of infrared radiation in the 1800s until 2014, was limited by the cost of the equipment. Development of semiconductors made the required technology available to mass industries. A concise historical overview of the development of thermography was given by Rogalski [1]. Now, IRT is probably the most applied method in temperature measurements. Industrial applications of this method range from medicine, metallurgy, automotive, aerospace, and the chemical industry to environmental studies [2,3]. IRT use in medicine and veterinary medicine for the detection of homeostatic imbalances that manifest as thermal differences is numerous. For example, orthopedic and bone or joint trauma, hormonal imbalance, vascular diseases, burns healing and detection of ulcers are just some of the diverse ongoing and growing studied applications of thermography [4]. Furthermore, IRT is regularly used in civil engineering to assess the structural health and energy efficiency of buildings [5,6]. Furthermore, thermography is a popular method of evaluation of historic monuments constructed of stone, brick masonry, or reinforced cement concrete [7]. In a similar manner, the method is used in material science for testing components. Next, IRT is used to search high-voltage nets, electrical installations and small-scale electronics for potentially dangerous hotspots that could lead to catastrophic failures, as detailed in [8]. Moreover, power plants are monitored by means of the IRT for optimal production, and wide geographic areas are monitored with IR cameras from an aerial point of view to detect geological properties or environmental damages.

Parameters which affect the accuracy of the thermal measurements are body/object emissivity, atmospheric molecular mixture, ambient temperature, wind, and distance from the target, external sources and any perturbation of the measured scene during the acquisition [9]. Emissivity is a measure of a targets ability to emit energy compared to a perfect emitter, a blackbody, which emissivity is one. This should be known to the observer in order to correctly set up the instrumentation. Atmospheric molecular mixture may absorb some energy radiated from the target so the IR camera does not detect the accurate thermal state of the target. In a similar manner, wind and ambient temperature could affect measured data. In addition, insufficient distance of an observer from the target may add to total thermal radiation detected by the IR camera due to observer’s reflection. Nevertheless, these factors are well known and should be accounted for by an experienced operator with sufficient knowledge of the physical background. In order to correctly determine the temperature, it is necessary to accurately determine the emissivity of the surface of the observed object, the reflected radiation of the environment, the ambient temperature, the relative humidity, and the influence of the wind, if it is strong, since the wind speed varies at different heights and it is easier to postpone the recording than to make a correction. In the case of outdoor images, it is convenient to perform the thermographic analysis when the sky is overcast, because this reduces the influence of the clear sky and negative values of the reflected temperature. When the exact emissivity and other parameters are not associated with the object, the displayed temperature is often referred to as the apparent temperature. If a higher emissivity is chosen, the temperature of the object will appear lower, and the opposite is also true. For these reasons, measurements are made on surfaces with high emissivity. If this is not the case, we use spray paints that increase the emissivity and allow accurate measurement. Humidity is important because water molecules scatter thermal radiation at greater distances. Ambient temperature is the effect of air molecules on the total heat flux. Wind contributes to the cooling of the surface, as does humidity, whose evaporation causes a lower temperature than the actual one displayed. In this contribution we discuss a novel factor, dangers of analyzing thermographs as images. The output of the thermal camera is an image, and the operations on that output allowed by the program support are the same ones usually applied to photographs. However, manipulation of the thermal camera output within the program support distorts (temperature) information about the target.

It should be noted that our study was conducted under specific conditions and may not be generalizable to all thermographic images or applications. For example, the type of object imaged, the distance between the object and the imaging system, and the ambient temperature may affect the accuracy of the temperature measurements. The choice of compression technique and the compression parameters used can also affect the quality of the compressed images.

## 2. Materials and Methods

**Figure 1:**
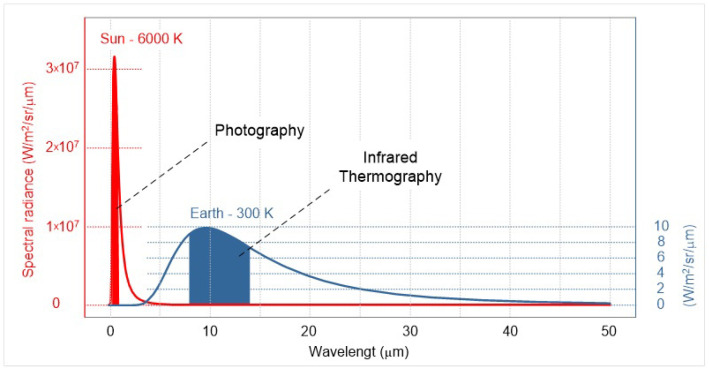
Comparison of the “amount” of radiation in the visible part of the spectrum and the detection range of the IR camera.

As previously mentioned, infrared thermography is a method of analyzing thermal radiation which essentially assigns a temperature designation depending on the characteristics and parameters of the environment. Therefore, in this contribution the term thermography refers to radiometric thermography which assigns a numerical value to the infrared thermal radiation. Note that this technical discipline is largely related to, but distinct from photography. In Figure 1, a comparison of the radiation power in the range of wavelengths that characterize those captured by cameras and IR cameras can be seen.

**Figure 2:**
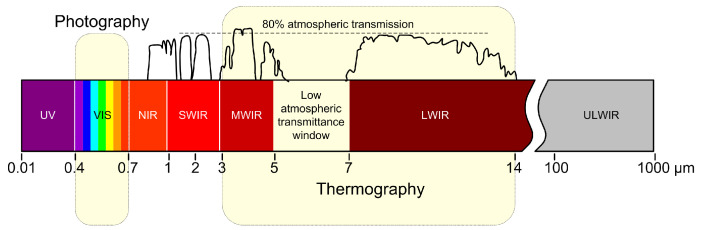
Classification of thermography in the field of electromagnetic radiation.

Both ranges in Figure 1 are electromagnetic radiation, and both obey the laws of optics. Nevertheless, thermal radiation can pass through opaque plastic objects, but not through glass, which is not an obstacle for visible radiation. The range of electromagnetic radiation most commonly associated with thermography is long-wave infrared (LWIR); the studies in this paper were conducted using a camera that detects radiation in the LWIR portion of the spectrum. Mid-wave infrared (MWIR) is associated with the analysis of higher-temperature objects that reach a maximum in this part of the spectrum due to the Wien displacement law [10], e.g., boilers, furnaces, and fire sources. Figure 2 shows the interval of electromagnetic radiation which is accessed by the means of thermography.

**Figure 3:**
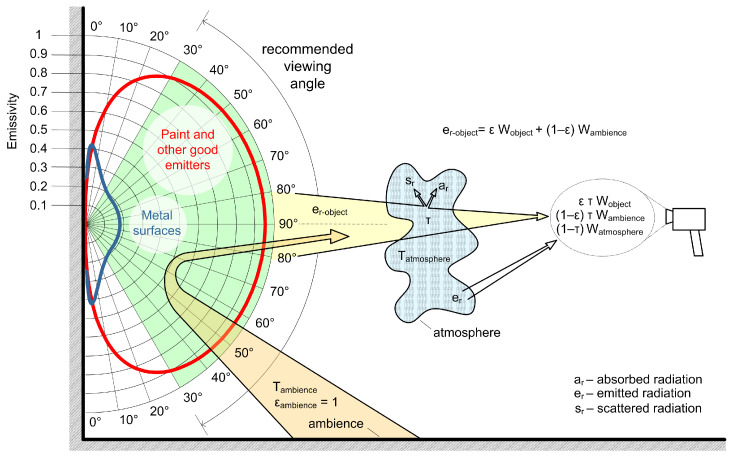
LWIR radiation detected by the IR camera.

The lenses of IR cameras are made of germanium which does not transmit the visible radiation and transmits only 50% of thermal radiation. The radiation that reaches the IR camera sensor is enough to change the resistance value of the measuring elements of the thermal sensor network and form the measured data. The temperature value is added to the measurement data based on the emissivity and calibration data. Hence, the IR camera does not measure the temperature, but the radiation in the infrared part of the spectrum, as shown in Figure 3.

**Figure 4:**
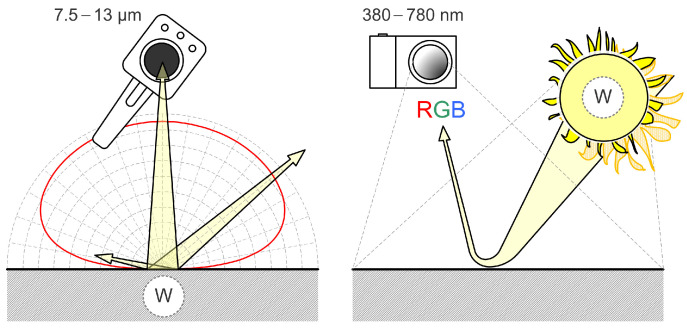
Comparison of thermography and photography.

The main difference between a photograph and a thermograph can be seen in Figure 4. As mentioned in the introductory part, objects radiate in the infrared part of the spectrum depending on their internal energy that determines their temperature and emissivity. Furthermore, emissivity depends on the surface treatment and physical properties of the material, while a photograph is created by the reflection of light (excitation) from the surface of the observed object, a thermograph is created by active long-wave radiation of the observed object (Figure 4).

The radiation of the object detected by the IR camera depends on the viewing angle, as shown in Figure 3 by red and blue curves of the emissivity dependent on the viewing angle. Smaller angles should be avoided as they lead to wrong conclusions for surfaces with lower emissivity. In addition to the radiation from the object, the environment also emits radiation which is added to the total radiation detected by the camera. At greater distances, the hydrous atmosphere enters the total energy balance and absorbs some of the radiation detected by the IR camera as a scattering contribution to the total radiation detected. To put it bluntly, thermographic analysis is not a simple method and depends on proper calibration of instruments and determination of all recording parameters which may affect the measured signal.

**Figure 5:**
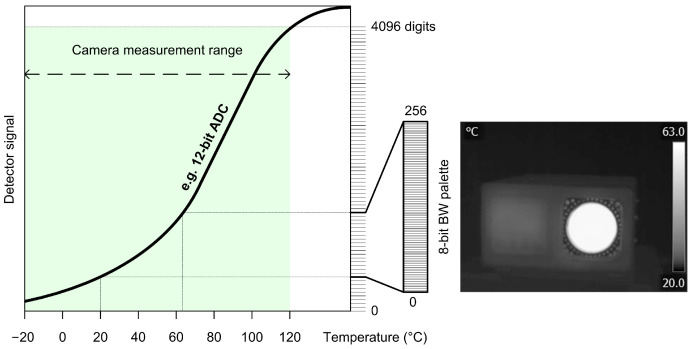
Conversion and display of the digital thermographic radiometric recording in image form.

IR thermographic systems typically employ a broad dynamic range, typically ranging from 12 to 16 bits [10,11]. The basic principle of analog-to-digital conversion (ADC) is illustrated in Figure 5. This process shares similarities between photography and thermography but also highlights a fundamental distinction. In photography, pixels represent one of 16.7 million colors, whereas in thermography, pixels, which can have 255 distinct levels, correspond to specific temperature values. In this particular case, there are 140 possible temperature values (ranging from −20 ${}^{{}^{\circ}}$C to 120 ${}^{{}^{\circ}}$C which represents the total number of temperature differences). In essence, Figure 5 depicts the conversion and portrayal of a digital thermographic radiometric recording in the form of an image.

**Figure 6:**
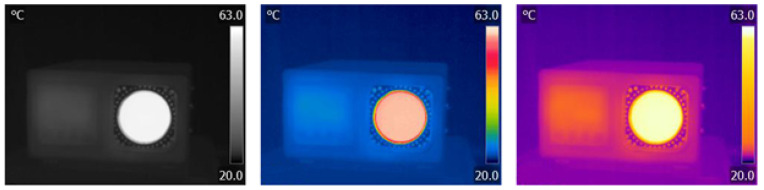
Blackbody thermographs displayed in three different palettes.

As thermographs, a priori, do not contain colors, the amount of information they provide is limited. Therefore, various palettes have been introduced to overcome the limitations of the instrument and provide the operator with as much information as possible about the object under study. Researchers often take advantage of palette features to obtain information based on individual channels that can only be accessed from RAW camera data [12]. Figure 6 shows the thermograph in three different color palettes. Although color palettes provide more information, the inverse BW palette has proven to be the best for detecting living bodies. Despite the fact that the information output from the IR camera looks like a photograph, it actually measures radiation over a much wider range of frequencies and does not distinguish colors. Images from infrared cameras are monochromatic because the cameras usually use an image sensor that cannot distinguish between the different wavelengths of infrared radiation. Objects radiate differently at certain wavelengths, which is not characteristic of photography because the light affects a much narrower range, as shown in Figure 1. Color image sensors require a complex design to distinguish wavelengths (Bayer filter, 50% G, 25% R, 25% B), and color has less significance outside the normal visible spectrum because the different wavelengths do not fit uniformly into the system of color vision used by humans.

**Figure 7:**
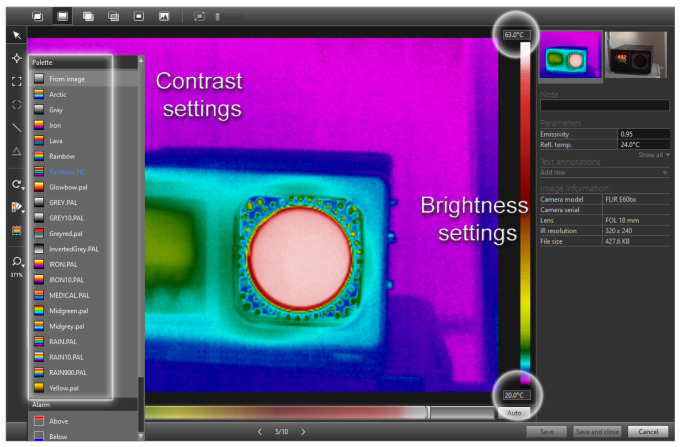
Dialog box of software support for infrared camera and functions that would correspond to the operations of adjusting brightness and contrast in photography.

The basic operations that are performed on images in this contribution are brightness and contrast. However, these operations on the thermograph change the information that the thermograph initially gives. Figure 7 shows a dialog box of the support program (FLIR Tools—basic software that comes with FLIR infrared thermal camera E60bx) that is provided with each camera, and what would correspond to the set brightness in thermography in photo processing, corresponds to the minimum and maximum temperature settings, i.e., the temperature space of the analysis. In terms of contrast, we have palettes that highlight individual areas of the temperature distribution with different hues and advertise the observation of changes in thermal patterns.

**Figure 8:**
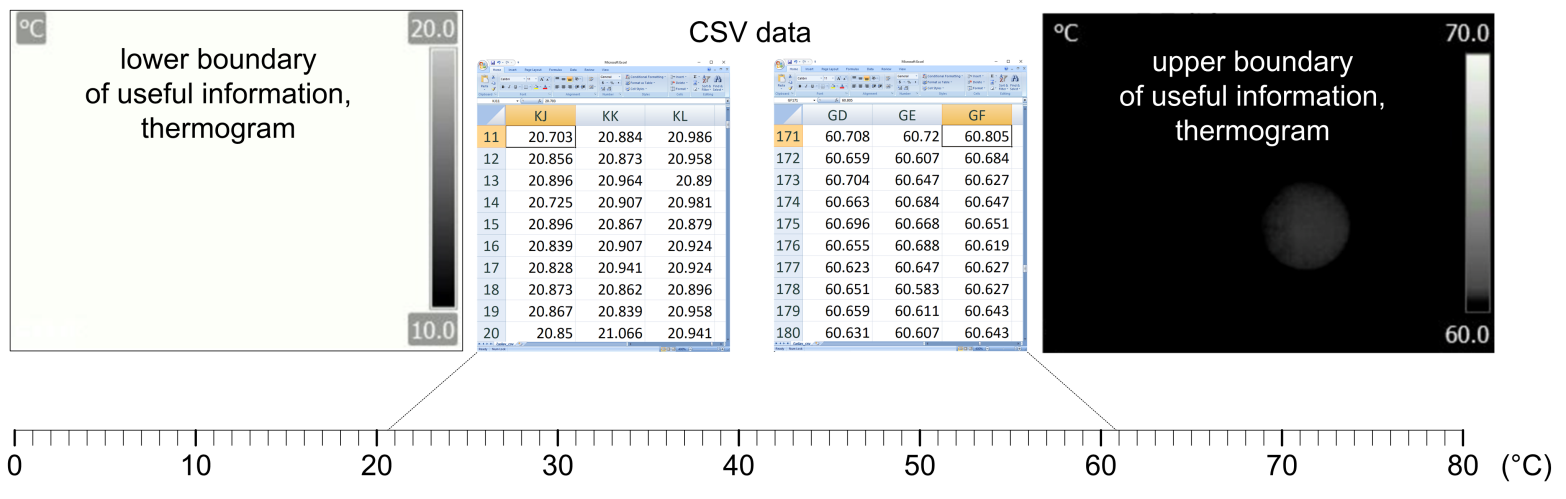
The temperature range of useful information and thermograms of the lower and upper limit.

When searching for raw data in CSV format, extreme values can be defined by searching for the minimum and maximum values. As can be seen in the description, Figure 8 shows the area of useful information on the thermograph. As can be seen on the left part of the image, the information does not exist at temperatures below 20 ${}^{{}^{\circ}}$C, and on the right part of the image it can be seen that the information disappears at temperatures above 60 ${}^{{}^{\circ}}$C, which can be seen by looking at the reference surface of the blackbody, whose temperature is 60.805 ${}^{{}^{\circ}}$C, in a barely perceptible way. The number of decimal places should not be interpreted as the precision of the measurement result, as it is a result of the statistical processing of AD, which includes the noise of semiconductor drift; for example, cameras that upscale thermal images have up to 12 decimal places.

**Figure 9:**
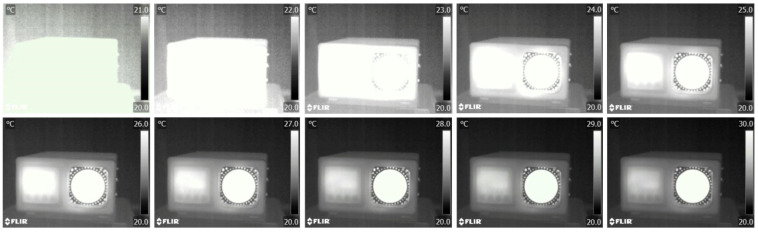
Display of increasing the temperature range by one degree ${}^{{}^{\circ}}$C from the lowest temperature.

Reducing the brightness of the thermograph in the software of the camera, starting with the smallest temperature value by changing the temperature range by one degree Celsius leads to an increase in useful information, but not above the reference value of the active surface of the blackbody. This is shown in Figure 9. Although the elements of the device and background are clearly visible on the thermograph, the value of the active surface is saturated and the actual information is not available. Therefore, it is limiting to only consider the display from the point of view of thermography.

**Figure 10:**
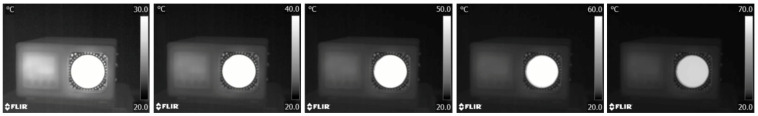
Display of increasing the temperature range by ten degrees ${}^{{}^{\circ}}$C from the lowest temperature to the maximum registered value.

Increasing the temperature range by ten degrees Celsius from the lowest temperature decreases the brightness, but increases the amount of useful information in relation to the visual experience of recognizing certain shades that correspond to temperature differences (Figure 10). Although the amount of information on the thermograph decreases visually, it increases from a thermographic point of view until the maximum recorded temperature is exceeded.

**Figure 11:**
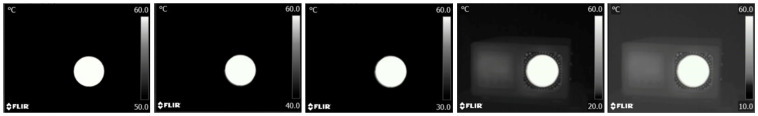
Display of increasing the temperature range by ten degrees ${}^{{}^{\circ}}$C from the highest temperature to the minimum registered value.

Applying the procedure to the maximum value and extending the range from the maximum value to the minimum value, as shown in the figure, the visual experience is not the same as in the previous figure, except for overlapping temperature intervals between 20 and 60 ${}^{{}^{\circ}}$C (Figure 11).

**Figure 12:**
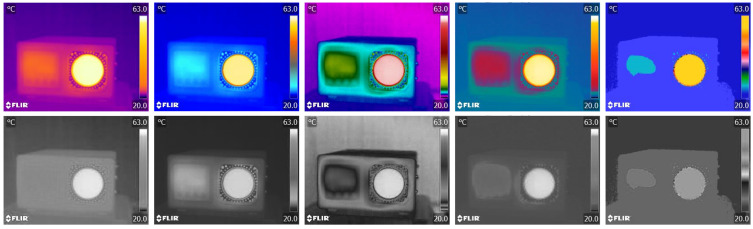
Thermographs in different palettes: Iron, Artic, Rainbow HC, Lava and Medical; and contrast comparison in the range of 255 shades of gray.

To summarize, from the above examples it can be deduced that in thermographic analysis, the most important parameter is to determine the minimum and maximum temperature in order to use the maximum dynamic range for information transfer. From a photographic point of view, the loss of information does not affect the visual impression, as can be best seen in the image in Figure 9, because the increase in the temperature range and the appearance of new information increases the contrast and recognizability of the analyzed object. On the other hand (Figure 10), the visual experience of the image changes significantly as the image goes into saturation due to the new information and becomes more difficult to recognize visually. Contrast in thermography is altered by the choice of color palette, as seen in Figure 12. To allow comparison, we transferred the thermographs from five different palettes (Iron, Artic, Rainbow HC, Lava, Medical) to BW images to show what the normalization of the histogram looks like when performed in the native thermogram, i.e., without color. The method is not well suited for practical use, because we can associate different temperatures in the image for certain shades, i.e., values, i.e., we lose information about temperatures.

### 2.1. Image Processing

**Figure 13:**
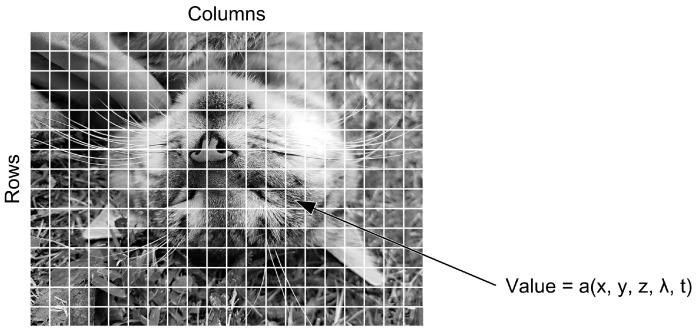
Digitization of a continuous image.

A digital image a[m,n] described in a discrete 2D space is derived from an analog image a(x,y) in a continuous 2D space by a sampling process often referred to as digitization. In the following, we present basic definitions related to digital image. The effect of digitization is shown in Figure 13.

The 2D continuous image a(x,y) is divided into *N* rows and *M* columns. The intersection of a row and a column is called a pixel. The value assigned to the integer coordinates [m,n] with m=0,1,2,…,M−1 and n=0,1,2,…,N−1 is a[m,n]. In most cases, a(x,y)—which is considered as the physical signal incident on the face of a 2D sensor—is actually a function of many variables, including depth (z), color (λ), and time (t) [13].

The image shown in Figure 13 has been divided into *N* = 16 rows and *M* = 16 columns. The value assigned to each pixel is the average brightness of the pixel, rounded to the nearest integer value. The process of representing the amplitude of the 2D signal at a given coordinate as an integer value with L different gray levels is usually referred to as amplitude quantization or simply quantization.

**Figure 14:**
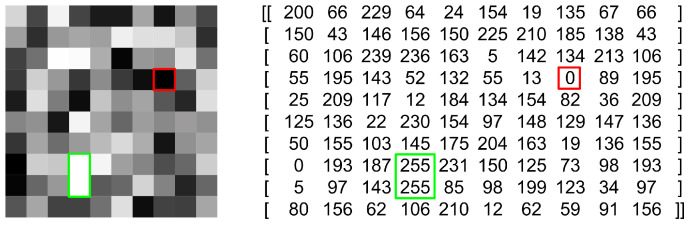
Quantized image with corresponding bit representation.

Quantization is the process by which the value of each pixel in the image is determined. First, a basic gray level, that is, the range of values that a pixel can have, is determined. Then, the value of the pixel in the digital image is calculated according to the value that comes from the continuous signal. If the gray level of an image is *n* bits, its gray level range has the value $2^{n}$. For example, the range of values that pixels can take for a gray level of 3 bits is [0–7], while the range of values that pixels can take for a gray level of 8 bits is [0–255]. The values that one pixel has is called the intensity of a pixel [14]. A quantized image is shown in Figure 14, where the red square encompasses the value 0 (black color), while the white color is represented by the value 255 encompassed by a green rectangle. All other colors (gray values) are in the interval 0–255.

### 2.2. Techniques Used for Image Processing and Compression

Three common image processing and compression techniques that are usually used in digital image processing are briefly discussed in the following.

The first technique is contrast and brightness adjustment, referring to changing the intensity and color palette of an image to improve its appearance or visibility. This is performed by changing the values of each pixel or by applying a curve function to the entire image. Adjusting the contrast and brightness can improve the quality of images that are too dark, too bright, or too dull.

The second technique minimizes the file size using JPEG compression, reducing the size of an image file by discarding some information non-important for a human eye perception. This technique uses a lossy compression algorithm which divides the image into pixel blocks and applies a discrete cosine transform (DCT) to each block.

The last image processing technique discussed is image interpolation. This technique creates a new image with a different resolution or aspect ratio from the original image by estimating the values of pixels that are not present in the original image. A mathematical function which takes into account the neighboring pixels and their distances from the new pixel, is applied. Image interpolation can be used to enlarge or reduce an image, or to correct geometric distortions such as rotation, scaling and perspective.

#### 2.2.1. Contrast and Brightness Adjustment

Digital image processing uses various algorithms and techniques to enhance, manipulate and analyze digital images. Brightness adjustment and contrast adjustment are two essential steps in image processing that can significantly enhance visual appeal of an image.

**Figure 15:**
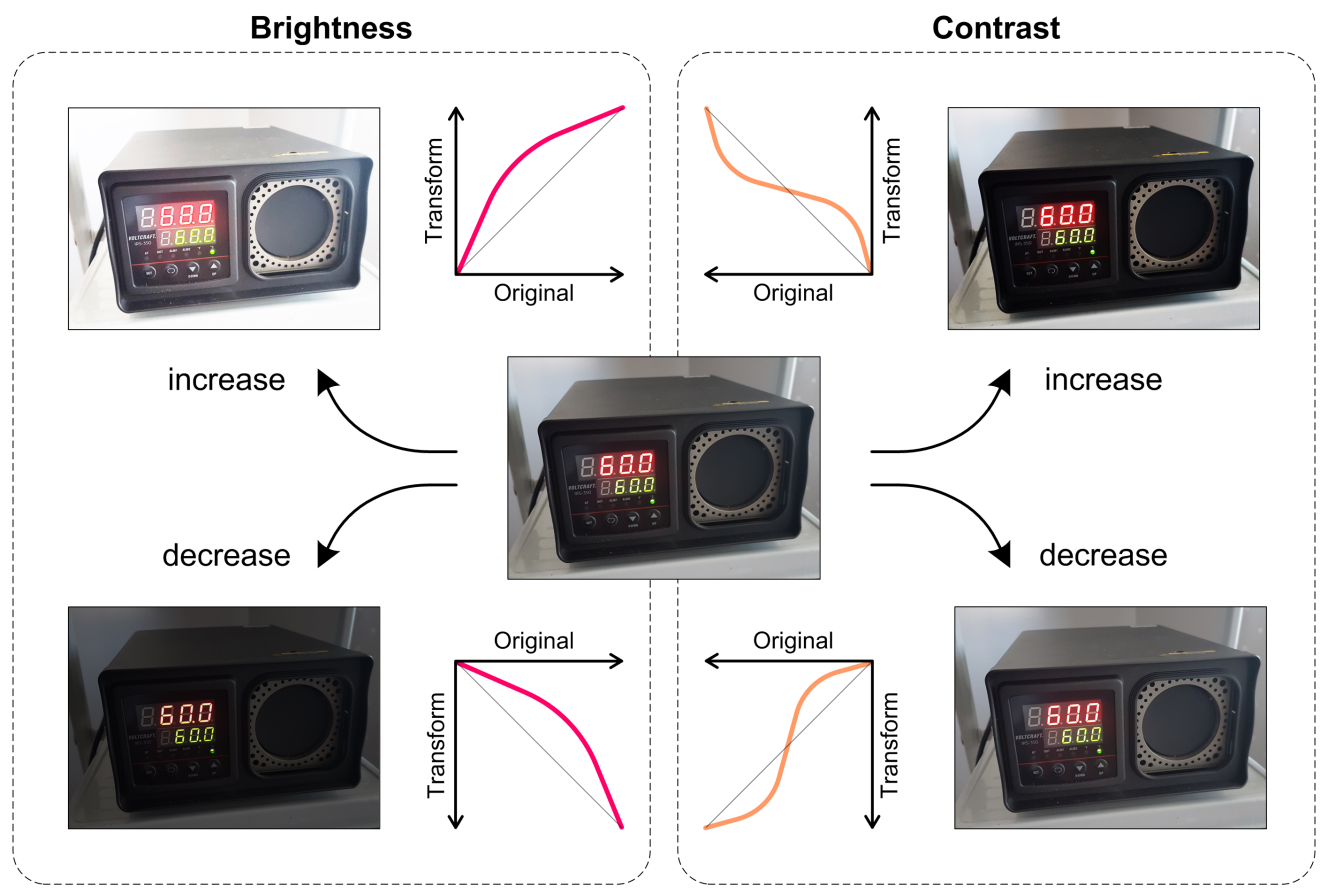
Contrast and brightness difference.

Brightness adjustment changes the overall brightness of an image, whereas contrast adjustment changes the difference in intensity between the light and dark areas of an image (Figure 15). The figure shows the non-linear transfer functions used to achieve the desired operations. Otherwise, they are standard image processing functions. Both adjustments can be used to improve the quality and visual appeal of an image, and to prepare it for further analysis or processing [15].

The adjustments mentioned above are achieved using a range of algorithms, including linear and non-linear. For brightness adjustment the following algorithms are used.

- Addition/Subtraction: Adding or subtracting a constant value to each pixel’s intensity value.
- Multiplication/Division: Multiplying or dividing each pixel’s intensity value by a constant value.
- Gamma Correction: Adjusting the image’s brightness using a non-linear gamma function.
- Histogram Equalization: Redistributing the pixel intensity values in an image to enhance contrast and brightness.
- Adaptive Histogram Equalization: A variant of histogram equalization that enhances the contrast of localized regions of an image.
- Logarithmic Correction: Adjusting the image’s brightness using a non-linear logarithmic function.
- Exponential Correction: Adjusting the image’s brightness using a non-linear exponential function.
- Piecewise Linear Transformation: Adjusting the image’s brightness using a non-linear piecewise linear function.

Algorithms for contrast adjustment are

- Histogram Stretching: Stretching the contrast of an image to improve its dynamic range.
- Contrast-Limited Adaptive Histogram Equalization (CLAHE): Enhancing the contrast of localized regions of an image while preventing over-enhancement and noise amplification.
- Sigmoid Correction: Adjusting the image’s contrast using a non-linear sigmoid function.
- Unsharp Masking: Increasing the contrast of an image by applying a high-pass filter to the image and adding the filtered result back to the original image.
- Tone Mapping: Adjusting the contrast and brightness of an image to reproduce the dynamic range of a high-dynamic-range (HDR) image on a low-dynamic-range display.

Among the algorithms for brightness adjustment, linear algorithms are usually used due to their simplicity and effectiveness. The addition/subtraction and multiplication/division algorithms are examples of linear algorithms in which a constant value is added or subtracted, or the intensity value of each pixel is multiplied or divided by a constant value. These algorithms adjust the brightness level of an image while maintaining the overall structure and contrast.

Similarly, histogram stretching is a linear algorithm commonly used for contrast adjustment. In this algorithm, the histogram of an image is stretched to increase its dynamic range, thereby increasing contrast and brightness. Histogram stretching is a simple and effective algorithm that maps the pixel intensity values of an image to a new range that can be specified by the user or calculated automatically [16].

Linear algorithms for brightness and contrast adjustment are preferred as they are easy to implement and understand, and can preserve the overall structure and contrast of an image. They provide a quick and easy way to adjust the brightness and contrast of an image while minimizing the risk of introducing unwanted artifacts.

#### 2.2.2. JPEG Compression

JPEG (Joint Photographic Experts Group) is a commonly used image compression standard, first introduced in 1992. The JPEG compression algorithm is based on the discrete cosine transform (DCT), a mathematical transformation that can efficiently convert an image into a frequency domain representation. The JPEG compression algorithm applies the DCT to the image, quantizes the resulting coefficients, and then encodes them using variable length coding [17].

One of the main features of the JPEG compression algorithm is its ability to achieve high compression ratios while maintaining reasonable image quality. The compression ratio can be adjusted by changing the quantization step size, which determines the level of detail preserved in the compressed image. Higher quantization steps result in higher compression ratios, but also a greater loss in image quality. JPEG compression is generally used in digital photography and similar applications where high compression rates are desired. However, it should be noted that JPEG compression is a lossy compression algorithm, meaning that some image information is discarded during the compression process. Consequently, repeated compression and decompression of JPEG images can result in a significant loss in image quality. Due to the introduction of a quantization error and the occurrence of artifacts in lossy compression, information that is emphasized by repeated compression is lost. To mitigate the loss in image quality, several variants of the JPEG compression algorithm have been developed over the years, such as JPEG2000, which uses a wavelet-based compression method and provides better compression efficiency and higher image quality than the original JPEG algorithm [18].

The JPEG compression algorithm uses a series of steps to compress an image. First, the image is divided into blocks of 8 × 8 pixels to allow for more efficient processing and analysis. For each block, the RGB values representing the red, green and blue color channels are converted to the YCbCr color space. This color space separates the image into a luminance component (Y) and two chrominance components (Cb and Cr) that capture the color information. Optionally, the chrominance components can be subsampled to reduce the amount of data needed to represent the color information. This step contributes to further compression by omitting some of the finer details in the chrominance channels, since the human visual system is less sensitive to color variations compared to luminance.

Next, the DCT is applied to each 8 × 8 block. This mathematical transformation converts the pixel values into a series of frequency coefficients that represent the spatial frequency components of the image. The DCT helps concentrate the energy of the image into fewer coefficients, allowing for more efficient compression. After DCT, the resulting coefficients are quantized using a quantization table. This table reduces the size of the coefficients by dividing them by certain values, effectively reducing their accuracy. Quantization discards some of the less important information in the coefficients, resulting in a controlled loss of image quality. The quantization table is critical to the trade-off between compression and image fidelity.

Once quantized, the DCT coefficients are encoded using Huffman coding. Huffman coding is a variable-length coding technique that assigns shorter codes to frequently occurring values and longer codes to less frequently occurring values. By exploiting the statistical properties of quantized coefficients, Huffman coding reduces the total bit rate required to display the image. Finally, the compressed image, consisting of the encoded quantized coefficients and the information required to reconstruct the image, is returned as the result of the JPEG compression process. This compressed representation can be stored or transmitted efficiently, resulting in a significant reduction in file size compared to the original uncompressed image.

**Figure 16:**
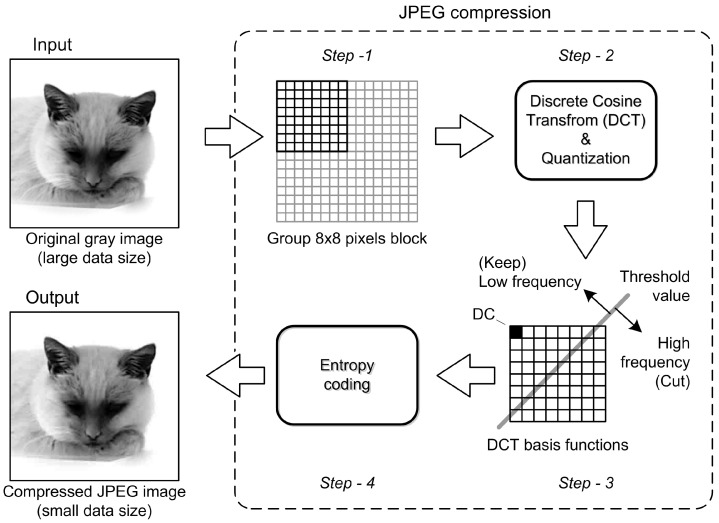
Algorithm and procedure of the JPEG image compression.

The JPEG decompression process reverses the above-mentioned steps to reconstruct an approximation of the original image. However, due to the lossy nature of JPEG compression, some information is irreversibly lost and some artifacts may appear in the decompressed image, such as blockiness, blurring, or ringing (Figure 16).

#### 2.2.3. Image Interpolation

Image interpolation is the process of estimating the values of pixels at locations other than the original ones. It is often used for resizing, rotating, or warping images. Image interpolation can be classified into two types: spatial domain interpolation and frequency domain interpolation. Spatial domain interpolation operates directly on the pixel values, whereas frequency domain interpolation transforms the image into a frequency representation and performs interpolation on the coefficients. Listed below are linear domain interpolation methods [19,20].

- Area-based interpolation. This is a resampling method using pixel area relation by computing the average pixel values of the pixels in the source image covered by each pixel in the destination image.
- Nearest neighbor interpolation. This method assigns the value of the nearest pixel to the new location. It is fast and simple, but it produces blocky and jagged images.
- Bilinear interpolation. This method uses a weighted average of the four closest pixels to estimate the new value. It is smoother than nearest neighbor interpolation, but it introduces some blurring and aliasing artifacts.
- Bicubic interpolation. This method uses a cubic polynomial function to fit the 16 nearest pixels and calculate the new value. It is more accurate and smoother than bilinear interpolation, but it is slower and more computationally intensive.
- Lanczos interpolation. This method uses a sinc function as a filter to compute the weighted average of a larger number of pixels. It preserves sharp edges and fine details better than bicubic interpolation, but it may introduce some ringing artifacts.

Some of the common frequency domain interpolation methods are mentioned below.

- Fourier interpolation. This method transforms the image into a Fourier spectrum and performs zero-padding or truncation to change the size of the spectrum. Then it applies an inverse Fourier transform to obtain the new image. It preserves the global information of the image, but it may introduce some Gibbs phenomena at edges and discontinuities.
- Wavelet interpolation. This method transforms the image into a wavelet representation and performs upsampling or downsampling on the coefficients. Then it applies an inverse wavelet transform to obtain the new image. It preserves the local features of the image better than Fourier interpolation, but it may introduce some artifacts due to aliasing or quantization.

**Figure 17:**
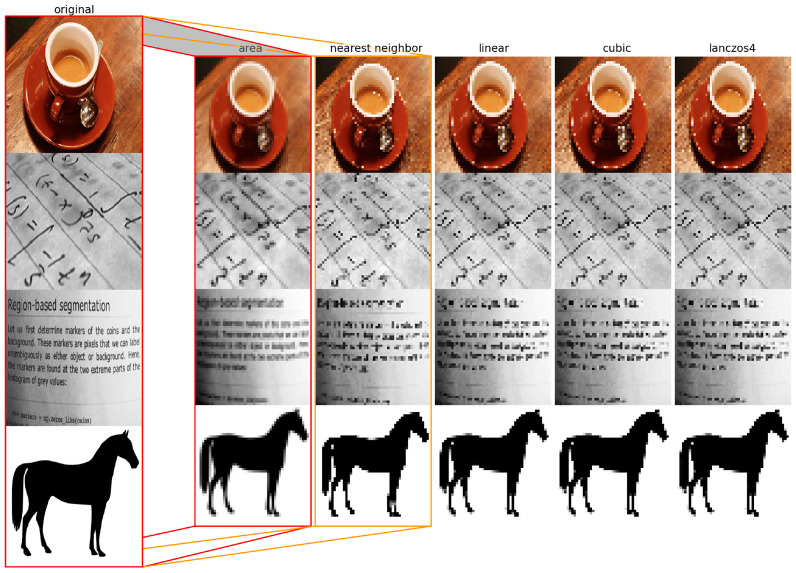
Image after downscaling with different interpolation methods.

The effects of the different interpolation methods are shown in Figure 17. The first column represents the original 400 × 400 resolution image, while the other columns show images reduced to 50 × 50 resolution using various interpolation methods shown in the title of each column. Degradation in image quality is clearly visible, but in a different way for each different interpolation method. This is an example of downscaling.

**Figure 18:**
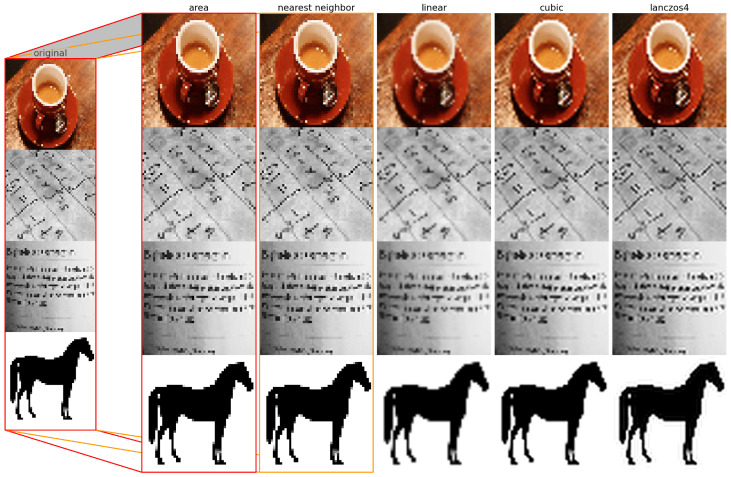
Image after upscaling with different interpolation methods.

The example of upscaling an image of low resolution (50 × 50) to a higher resolution (400 × 400) can be seen in Figure 18.

From Figure 18, it can be seen that two out of the five previously mentioned methods do not add blur to the images, while linear, cubic and lanczos try to smooth the missing data by adding blur.

### 2.3. Thermography

The IR camera used in this study was calibrated prior to the analysis by the blackbody set to an appropriate temperature. The reasoning and requirements for performing the calibration are given in this section.

### 2.4. Radiometric Data Collection and Analysis

**Figure 19:**
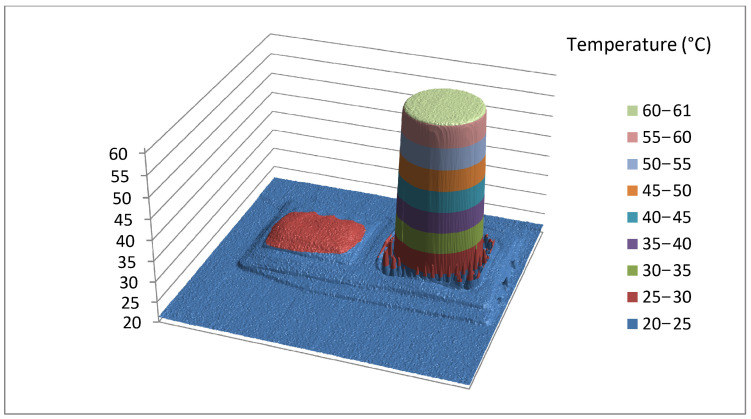
Thermograph of a blackbody.

**Figure 20:**
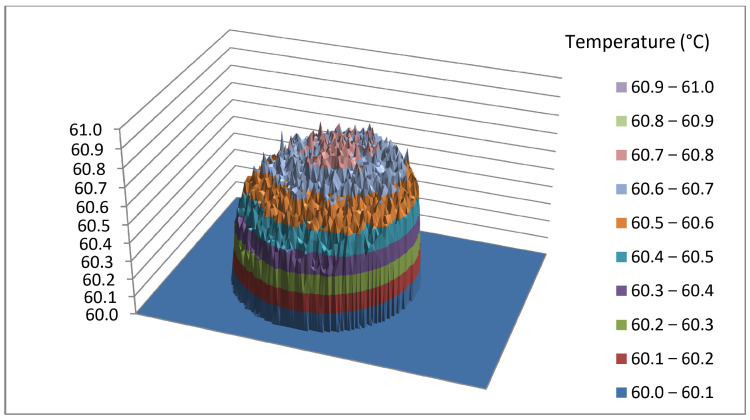
Thermograph of the active part of the emitting surface.

Data which an IR camera provides to the user can be further processed in the program support included with the camera. Within the program it is possible to make corrections and alter the parameters that are determined incorrect during the shooting. For example, changing the palette and thus increasing the contrast or changing the temperature range results in a lighter or darker display of the thermograph. Most importantly, all data can be exported to CSV files which can be used for further numerical analysis. The CSV file exported in this way is not the raw data of the camera, but contains the influence of all recording parameters. However, due to the large number of decimal places that are the result of statistical processing, the given data can be considered as a reference value, especially if it shows a reference radiation source. Figure 19 shows the analysis of the thermographic image of the entire blackbody instrument; while Figure 20 shows only the active part of the emitting surface, isolated, where the influence of NETD is best seen and all manipulations with the thermogram will mainly affect small temperature differences. Despite the accuracy of the camera, which is ±2 ${}^{{}^{\circ}}$C or ±2% of the measured value, the data shown have a relative accuracy defined by the NETD (noise equivalent temperature difference) of 45 mK, while the absolute value of the whole matrix can be corrected by contact measurement or by deviation from the mean value of the blackbody reference surface.

Note that Figure 19 shows a 3D graph of the data extracted from the CSV file. It is a juxtaposition of the thermograph and the active part. The blackbody was appropriately set at a temperature of 60 ${}^{{}^{\circ}}$C. However, it can be seen in Figure 20 that the measured temperature on the substrate is between 60.5 and 60.9 ${}^{{}^{\circ}}$C. The deviation observed can be attributed to the surface roughness of the object being observed. It is important to note that this deviation is typically not a result of temperature differences, but rather stems from variations in radiation caused by the roughness of the object’s surface. This roughness causes the object to emit radiation at different angles with respect to the normal of the thermal camera lens. This phenomenon further reinforces the understanding that infrared (IR) measurements are not solely capturing temperature but rather thermal radiation, to which the temperature values are assigned based on the calibration data. By recording the reference radiation source, we have provided a reference data set that will serve us for hazard analysis when thermographic radiometric data is treated as an image.

### 2.5. Accuracy of Temperature Measurement in Thermography

A thermograph as source information about thermal radiation obtained with an IR camera is only as good as the quality of the camera. Usually we associate the quality of a photo camera with the resolution, which is 6000 × 4000 pixels on average, but this is not the case in thermography. Resolution helps in better observation of details, but not in the precise determination of radiometric data. The current standard value for an IR camera resolution is 640 × 512. The maximum resolution available for civil applications is 2560 × 2048 which comes from InfraTec ImageIR 9400 [21]. In technical applications, a minimum resolution is often specified; for example, the minimum resolution for photovoltaic panel analysis is 320 × 240 with mandatory photo capture according to [22]. Use of an IR camera with a resolution larger than 320 × 240 pixels in combination with a separate photo camera is recommended. In many cases, the basic photo camera integrated into the infrared camera is not capable of providing the desired resolution. For an IR camera with 640 × 480 pixels, a separate photo camera with at least 9 Mpix is suitable. That said, probably the most important specifications are the NETD and accuracy (${}^{{}^{\circ}}$C). NETD defines the smallest temperature difference that can be detected between two adjacent sensors (pixels). For quality cameras, NETD is 20–30 mK, and the minimum value on the market is 15 mK. For this study, the FLIR E60bx camera with an NETD of 45 mK was used (Table 1). The accuracy (${}^{{}^{\circ}}$C) of the used FLIR camera is ±2${}^{{}^{\circ}}$C or ±2% of the measured value. The specified value represents a significant difference when thermography is used for medical purposes [23]. If the measured value is 38 ${}^{{}^{\circ}}$C instead of 36${}^{{}^{\circ}}$C, this makes a huge difference in medical applications. Therefore, thermographic examinations are accompanied by a contact temperature measurement, which helps to find a reference value. Precise and accurate contact thermometers are more expensive than blackbodies, which are the reference standard in the infrared thermography. A blackbody is a reference radiation source against which the moderation of the camera is checked, but only if the blackbody is moderate. Figure 15 shows the Voltcraft IRS −350 set to 60 ${}^{{}^{\circ}}$C and used to calibrate the FLIR IR camera.

Specifications of the blackbody used in the analysis are presented in Table 2.

It can be seen from Table 2 that the accuracy of the blackbody (±0.5 ${}^{{}^{\circ}}$C) is higher than the accuracy of the FLIR IR camera (±2 ${}^{{}^{\circ}}$C). For this reason, it is recommended to perform a calibration of the IR camera once a year and check the deviation from the reference value. Table 3 shows the difference in the temperature of the blackbody and the temperature which the FLIR camera is detecting.

Temperature differences presented in Table 3 are affected by the IR camera and blackbody accuracies. Since these are two independent quantities, accuracy can be expressed as the square root of individual influences. From Table 1 it is clear that the accuracy of the camera used is in the range of ±2 ${}^{{}^{\circ}}$C. To perform the base correction, we use the blackbody as a reference. With the reference surface setting of 60 ${}^{{}^{\circ}}$C and the temperature range in which the blackbody has an accuracy of ±0.5 ${}^{{}^{\circ}}$C according to Table 2, the camera shows an average temperature of 0.6 ${}^{{}^{\circ}}$C higher in the observed temperature interval from 50 ${}^{{}^{\circ}}$C to 90 ${}^{{}^{\circ}}$C (Table 3). This means that the values shown by the camera can be reduced by the specified value in order to correct for introduced measurement error which will be seen from the detailed analysis of the CSV reference data set. As these are two independent values, the composite uncertainty can take on values up to 0.78 ${}^{{}^{\circ}}$C if the largest amount of blackbody deviation is taken into account. For reference, cheaper models of IR cameras have an error of 4.61 ${}^{{}^{\circ}}$C, and uncalibrated cameras up to 6.46 ${}^{{}^{\circ}}$C [24]. Calibration of an IR camera, which is initially performed at the factory, is performed at one of the reference temperatures for all individual elements of the sensor. Additionally, factory calibration is performed for different temperature values (Table 3), and the values are written within the program support of the camera. For the FLIR E60bx camera, the support operates on a Windows CE system.

## 3. Results

### 3.1. Presentation of the Findings from Each of the Three Analyses

**Figure 21:**
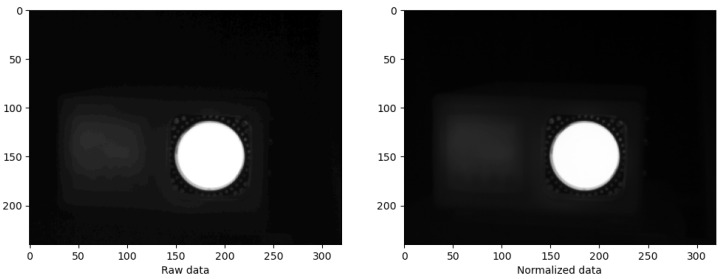
Comparison of raw and normalized data.

The study involves obtaining an image from a CSV file consisting of 240 rows and 320 columns of data. The CSV file contains numerical values representing temperatures ranging from 20.703 ${}^{{}^{\circ}}$C to 60.805 ${}^{{}^{\circ}}$C. The process of creating an image from the data requires reading the data from the CSV file into a program, manipulating it as required, and then presenting it in a visual format that can be viewed as an image. To access the available data, a comparison between raw data and normalized data (values 0–255) is presented in Figure 21.

As seen in Figure 21, a subtle difference between the two images exists. The raw data has the effect of streaking, which can be noted in the leftmost part of the image, while the same area is smoothed on the image presented with normalized data. All image processing are performed with the normalized data.

### 3.2. Image Processing

**Figure 22:**
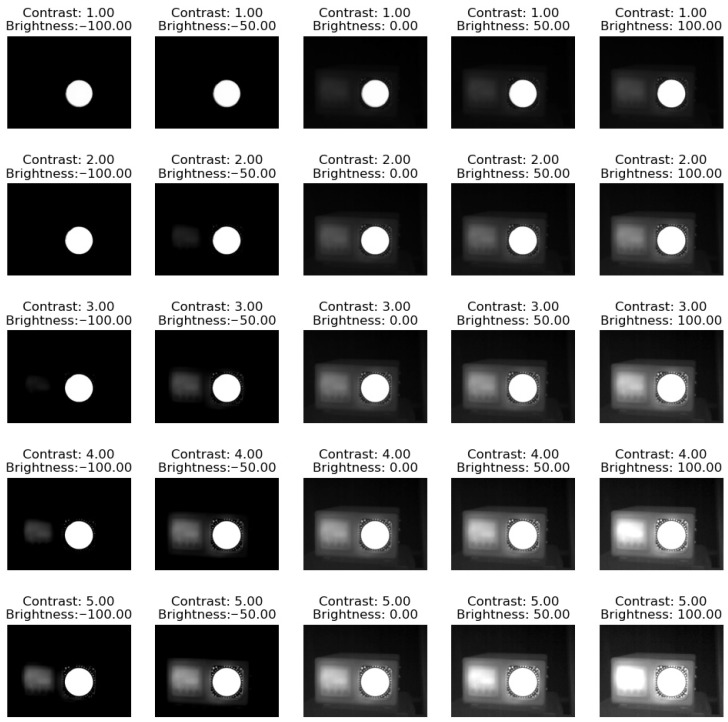
Results of brightness and contrast manipulation.

Let us denote u(i,j) as the normalized data, α as the contrast-modifying factor, and β as the offset for regularizing brightness. A formula can be created that changes the contrast and brightness accordingly (Equation (1)), and the resulting data would be contained in f(i,j):

(1) f(i,j)=α·u(i,j)+β,

where α ranges from 1 to 5 in order to change the contrast, and β has values from −100 to 100, thus providing multiple options. The results are shown in Figure 22, and they clearly show that changing the brightness and contrast of the image can have a significant impact on the readability of the data in the image. Increasing the brightness can cause the lighter areas of the image to appear brighter and the darker areas to appear lighter, potentially making some of the temperature differences less visible. Similarly, increasing the contrast can make the temperature differences between adjacent areas more apparent, but it can also cause the image to appear more saturated and overemphasize small temperature differences. Therefore, it is important to carefully adjust the brightness and contrast of thermographs to optimize their readability without distorting the underlying temperature data.

### 3.3. Image Compression

As the FLIR uses JPEG compression, we analyze how much the compression ratio of the JPEG compression algorithm affects the underlying temperature data. We examine the effects of lossy and lossless image compression on objective image analysis. Our analysis begins by importing raw image data into our programming environment. We then applied both lossy and lossless image compression techniques to the images in our data set. For lossless compression, we used PNG compression as it preserves the original image data without the loss of information, while for lossy compression, we used JPEG.

For the purpose of objective image analysis, several metrics are used in this study, including AAE, MSE, PSNR, and SSIM. These metrics are used to quantify various aspects of image quality and to evaluate the effects of different image compression techniques on the accuracy of objective image analysis.

Average absolute error (AAE) is a measure of the average difference between the pixel values in the original and compressed images. The mean squared error (MSE), calculates the average of the squared differences between the pixel values in the original and in the compressed image. The peak signal-to-noise ratio (PSNR) measures the ratio between the maximum possible signal strength and the amount of noise in the image. The structural similarity index (SSIM) is a metric that considers the structural similarities between the original image and the compressed image. These metrics are detailed in [25].

**Figure 23:**
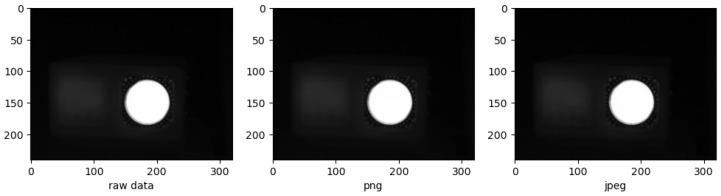
Comparison of the raw data, PNG, and JPEG compression.

The compression of raw data using the PNG and JPEG compression algorithms are shown in Figure 23. JPEG compression is performed with the image quality parameter set to 90 while the PNG compression is lossless.

At this level of detail and these compression ratios, the difference is not discernible. Therefore, quantifiable and comparable results must be obtained in order to be used for the evaluation of the effectiveness of different image compression techniques for objective image analysis. The metrics are listed in Table 4.

One might wonder why metrics such as MSE, PSNR, AAE, and SSIM are used when PNG compression is considered lossless. Although PNG compression is lossless, it still involves quantization of the raw image data. Quantization refers to the process of approximating the continuous values of the image pixels by a finite set of values. This process introduces some error into the compressed image data, even though it is considered lossless.

**Figure 24:**
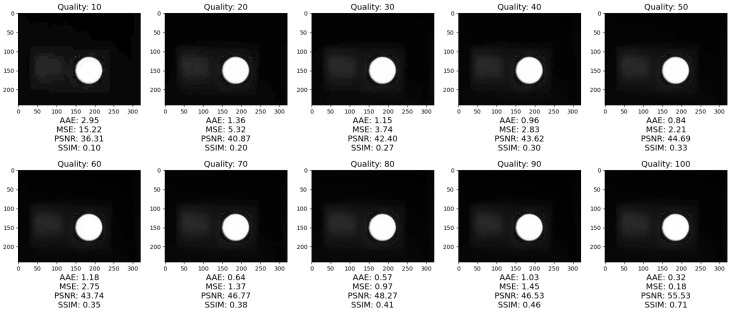
Results of JPEG compression with various compression ratios.

The objective image analysis is performed on ten different compression ratios of the JPEG compression algorithm. The image quality factor is set from 10 to 100 in increments of 10. The results of the compression can be seen in Figure 24.

**Figure 25:**
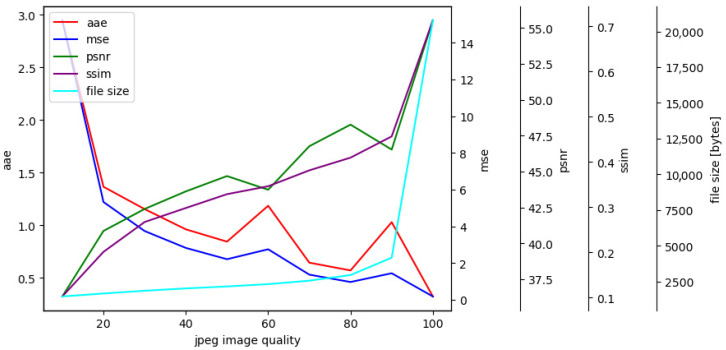
Objective evaluation of image quality in JPEG compression.

Objective image quality assessment for JPEG compression for various degrees of JPEG image quality are shown in Figure 25.

Increasing the JPEG quality parameter generally improves the quality of the compressed image. The AAE and MSE decrease with an increase in the JPEG quality parameter, while PSNR and SSIM increase, as higher quality JPEG images have less artifacts and distortions and retain more details from the original image. However, it should be mentioned that increasing the JPEG quality parameter beyond a certain point does not significantly improve image quality, but instead increases file size. Therefore, a balance between image quality and file size when selecting the optimal JPEG quality parameter should be achieved.

**Figure 26:**
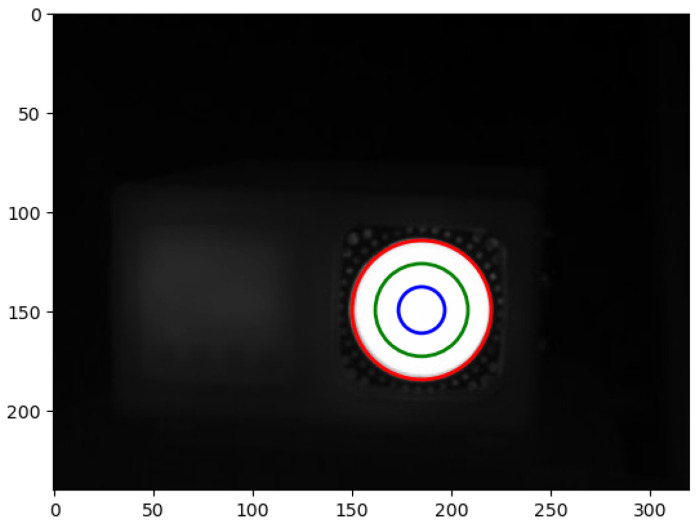
Defining the region of interest of the blackbody.

To detect the change in values in thermography with the decrease in image quality, a region of interest (ROI) must be defined. In Figure 26, three ROIs are defined, encompassing the reference region of the blackbody with the temperature value of 60 ${}^{{}^{\circ}}$C.

Zones with their respective ROI radiii are:

- Zone 1 (red circle)—full radius (includes the entire radiating area);
- Zone 2 (green circle)—2/3 of the radius;
- Zone 3 (blue circle)—1/3 of the radius.

**Figure 27:**
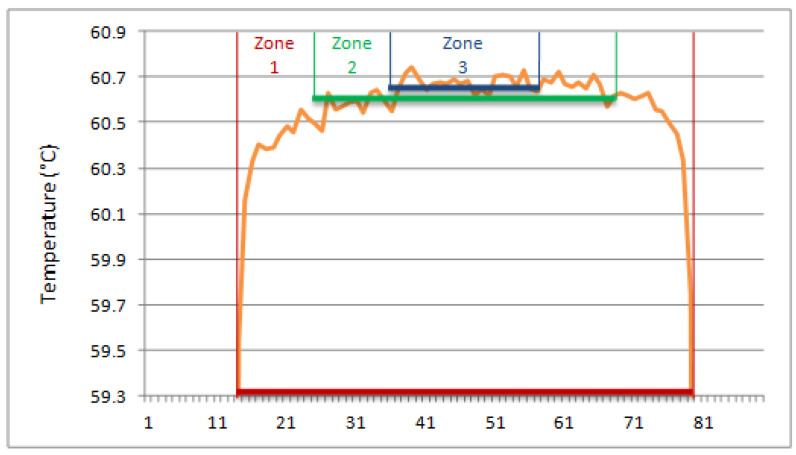
Linear temperature pattern and corresponding zones.

The reason behind opting for such zone diameters is depicted in Figure 27. Selection of the ROI radius is based on one of the linear temperature patterns.

Two metrics are calculated to evaluate the blackbody misrepresentation, mean and median:

- Mean, also known as the arithmetic mean, is calculated by adding all the values in a data set and dividing the sum by the total number of values. It is the most commonly used measure of central tendency and is often used to describe normally distributed data.
- Median is the middle value in a data set when the values are arranged in numerical order. It is the value that separates the top half of the data set from the bottom half. The median is useful when the data set contains extreme values or outliers that can distort the mean.

Values of the mean and median for the three circles are presented in Table 5.

**Figure 28:**
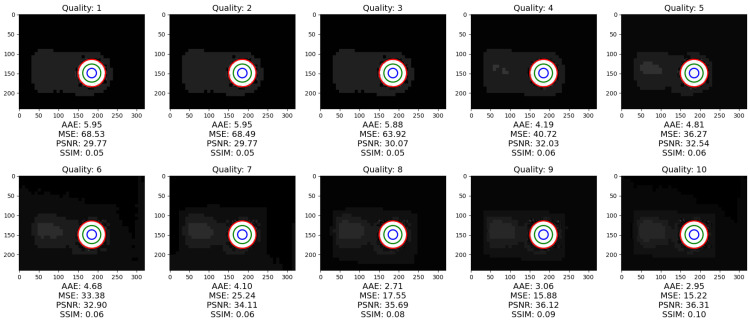
Results of compression with highlighted ROIs.

The median values in Table 5 are equal, but the mean values change with the diameter of the circle. For the best results, the following experiments use the ROI, defined by the blue circle (Zone 3) in Figure 25. Since the experiments previously shown in Figure 24 did not show much degradation, a higher compression ratio was used to test the hypothesis that the temperature value of the ROI changes with different compression ratios. The results are shown in Figure 28. All zones are depicted and the AAE, MSE, PSNR, SSIM are calculated. Since the ROIs cover a small range with mostly the same values, the results are similar.

For all measurements seen in Table 6 we can conclude that the mean and median temperature values are the same for the observed ROIs. Therefore, in Table 6 only the mean values are compared to the baseline values presented in Table 5.

**Figure 29:**
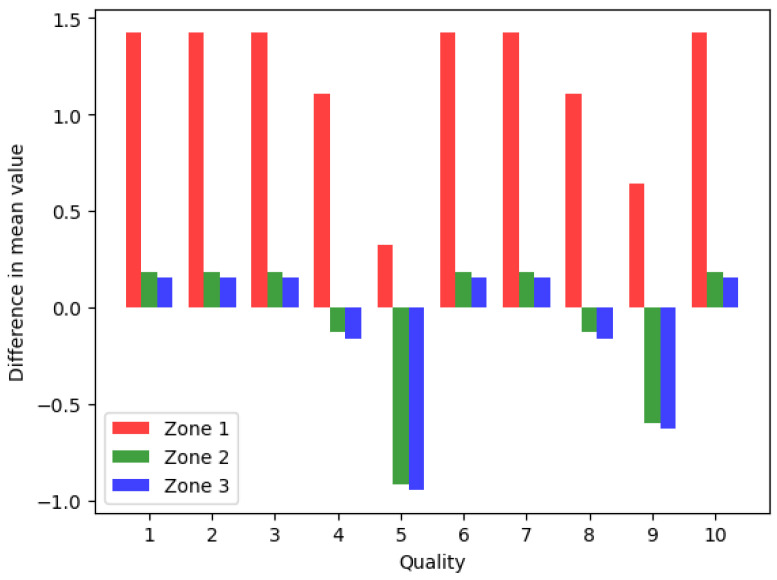
Differences in the mean values of the three observed zones.

The differences in the mean values presented in Table 6 are shown on Figure 29.

From the perspective of classical image processing, all objective image quality metrics change in the correct order with increasing image quality factor, i.e., AAE and MSE become smaller, while PSNR and SSIM become larger. Since the ROI is quite small, the changes in temperature values are minimal, but they convey the message that the data values are distorted when using classical image processing methods and therefore cannot be used. Table 6 clearly shows how compression affects the temperature readings, resulting in a non-negligible difference.

### 3.4. Image Interpolation

Since the values in the interface are not equal (Figure 20), a need to enlarge the area for detailed analysis arises. Therefore, in this section we present the blackbody area enlarged to capture the degradation of the data at a new level.

**Figure 30:**
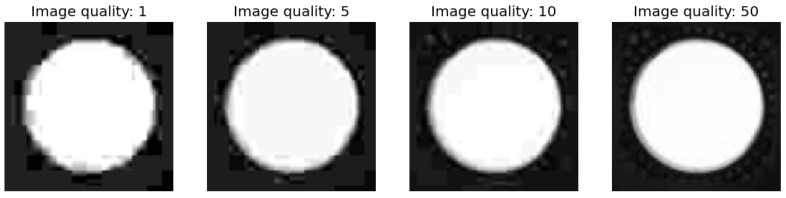
Enlarged portion of the image showing the blackbody at different compression ratios.

**Figure 31:**
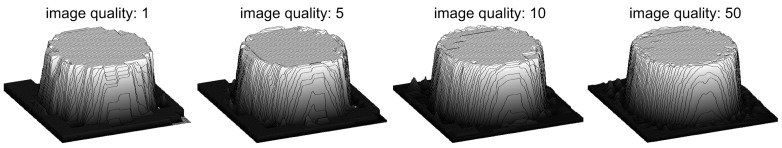
3D representation of the blackbody at different compression ratios.

Image interpolation is a method of estimating unknown pixel values between the known pixels of an image, and for this case a sufficiently degraded images are required, so the images encoded with image quality factor 1, 5, 10 and 50 are used for the following experiments (Figure 30) and their 3D representation is shown of Figure 31.

**Figure 32:**
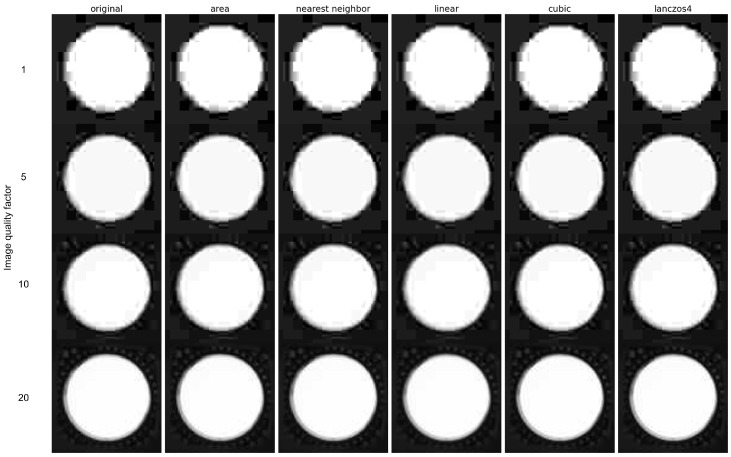
Results of image interpolation with different methods.

We observe that the edges have deteriorated, and it is in this area that the greatest discrepancy with the original image is seen. Figure 32 shows the upscaling from the original image resolution 90 × 90 to a resolution of 720 × 720 in the hope of obtaining a clearer edge of the blackbody.

## 4. Discussions and Conclusions

Despite the fact that thermography is a widely used method for measuring temperature variations on the surface of objects, the large amount of data generated by IR imaging systems can be difficult to manage and store especially with the continuous growth of the resolution of thermograms. To solve this problem, we have explored the use of image compression techniques, specifically JPEG compression, to reduce file size while maintaining image quality.

We manipulated brightness and contrast to visually assess how they affect the underlying data. It was found that brightness and contrast balance are required to preserve the original data as too much brightness or too hard a contrast can change the visual representation of the temperature readings.

To investigate the effects of image manipulation on thermographic images, we used a blackbody as a reference and defined three ROIs at different radii around the blackbody. We then varied the JPEG quality parameters to compress the images and evaluated the effects on the temperature values in the ROIs. It was found that decreasing the JPEG quality parameter resulted in decreased image quality and vice versa but only up to a certain point (75–80 image quality index), where further increasing the quality parameter did not significantly improve image quality. This is consistent with previous studies that have shown a trade-off between file size and image quality with JPEG compression [26]. Interestingly, we found that the mean and median temperature values of the ROIs were not significantly affected by the decrease in JPEG quality, except for the largest ROI, where the mean temperature value decreased slightly at higher compression rates. This suggests that JPEG compression can be applied to thermographic images without significantly affecting temperature values in specific ROIs.

In addition, we conducted experiments on image interpolation, where we found that upscaling the images did not result in clearer edges which are necessary for a correct reading of the temperature parameters.

Infrared thermography is related to temperature measurement, although it is essentially a method of measuring thermal radiation to which temperature information is added based on the recording parameters. The result of the measurement is an image which represents numerical values indirectly and in some way hides the measurement data. Almost every software that comes with a camera for processing the recorded thermograms allows data to be exported to CSV. The CSV file is not a RAW file of the camera sensor, but processed data that includes corrections for reflected radiation and emissivity.

By visualizing the CSV data sets, we can observe the different behavior of individual elements of the matrix of temperature data sets and the accuracy with which the exact temperature value can be obtained. Deviations of individual numerical values are not expressed in the thermograph, so visualization of the measured data is presented in a spreadsheet (Excel) and the corresponding program supplied with the camera. Although the output of the measurement data is an image, the operations that we apply to photographs cannot be applied to it without distorting the information about the measurement data.

Using a thermograph as an example, we showed all the editing possibilities, as well as the effects that individual actions have on the visual representation of the measurement results. The software in the background itself performs the interpolation of the thermograph into a larger image without the possibility of viewing the computer background of the process. Unlike photography, which is stimulated by light and external stimuli, thermography is based on the radiation of an object’s internal thermal energy. The conversion of raw data from the photon-counting camera sensor into a digital form is similar to processing information about the change in resistance due to thermal energy, with the difference that there is no color in thermography.

To improve the contrast and visibility of the different thermal patterns, palettes are introduced. In essence, it is a black and white recording that can be treated like a photograph, but it is always pointed out that it is measurement data presented in the form of an image and not a photograph. To prove the previously mentioned hypothesis, we used the CSV data set as a reference and performed standard forms of image transformation. Considering the physical behavior of the radiation distribution on the reference surface, we segmented the space into three ROIs and performed numerical evaluation. The expected deviations of individual zones are less surprising than the storage of the image under quality five, which clearly indicates the distortion of the radiometric data due to the compression.

## Figures and Tables

**Table 1:** Specifications of the E60bx FLIR camera.

Specification	Value
IR resolution	320 × 240
NETD	45 mK
FOV	25° × 19°
IFOV	1.36 mrad
Spectral range	7.5–13 µm
Temperature range (${}^{{}^{\circ}}$C)	−20 to +120
Accuracy (${}^{{}^{\circ}}$C) or (%) of reading	±2 ${}^{{}^{\circ}}$C or ±2%

**Table 2:** Table of features of the Voltcraft IRS -350 used for imaging and analysis.

Settings	Value
Temperature range	50 ${}^{{}^{\circ}}$C to 350 ${}^{{}^{\circ}}$C
Accuracy	±0.5 ${}^{{}^{\circ}}$C at 100 ${}^{{}^{\circ}}$C
	±1.2 ${}^{{}^{\circ}}$C at 350 ${}^{{}^{\circ}}$C
Stability	±0.1 ${}^{{}^{\circ}}$C at 100 ${}^{{}^{\circ}}$C
	±0.2 ${}^{{}^{\circ}}$C at 350 ${}^{{}^{\circ}}$C
Emissivity of measuring area	0.95
Operating temperature	5 ${}^{{}^{\circ}}$C to 35 ${}^{{}^{\circ}}$C

**Table 3:** Results of the calibration of the IR thermal camera E60bx.

IRS-350 Blackbody (${}^{{}^{\circ}}$C)	FLIR E60bx (${}^{{}^{\circ}}$C)	Temperature Differences
50	50.7	0.7
60	60.5	0.5
70	70.6	0.6
80	80.8	0.8
90	90.6	0.6
100	101.0	1.0
110	110.8	0.8
120	121.0	1.0
130	131.0	1.0
140	141.0	1.0
	Average:	0.8

**Table 4:** PNG and JPEG comparison.

Metric	PNG	JPEG
AAE	0.25	1.03
MSE	0.08	1.45
PSNR	58.86	46.53
SSIM	0.82	0.46

**Table 5:** Mean and median values for the ROIs.

Region of Interest	Temperature (${}^{{}^{\circ}}$C)	
	Mean	Median
Zone 1	59.38	60.49
Zone 2	60.62	60.65
Zone 3	60.65	60.65

**Table 6:** Mean and median values for the ROIs.

Quality	Mean (${}^{{}^{\circ}}$C)			Baseline Mean (${}^{{}^{\circ}}$C)			Difference (${}^{{}^{\circ}}$C)		
	Zone 1	Zone 2	Zone 3	Zone 1	Zone 2	Zone 3	Zone 1	Zone 2	Zone 3
1	60.805	60.805	60.805	59.38	60.62	60.65	1.425	0.185	0.155
2	60.805	60.805	60.805	59.38	60.62	60.65	1.425	0.185	0.155
3	60.805	60.805	60.805	59.38	60.62	60.65	1.425	0.185	0.155
4	60.490	60.490	60.490	59.38	60.62	60.65	1.110	−0.130	−0.160
5	59.704	59.704	59.704	59.38	60.62	60.65	0.324	−0.916	−0.946
6	60.805	60.805	60.805	59.38	60.62	60.65	1.425	0.185	0.155
7	60.805	60.805	60.805	59.38	60.62	60.65	1.425	0.185	0.155
8	60.490	60.490	60.490	59.38	60.62	60.65	1.110	−0.130	−0.160
9	60.019	60.019	60.019	59.38	60.62	60.65	0.639	−0.601	−0.631
10	60.805	60.805	60.805	59.38	60.62	60.65	1.425	0.185	0.155

## Data Availability

The data presented in this study are available on request from the corresponding author.
